# CCHFV vaccine development, current challenges, limitations, and future directions

**DOI:** 10.3389/fimmu.2023.1238882

**Published:** 2023-09-11

**Authors:** Büşra Ahata, Günseli Bayram Akçapınar

**Affiliations:** ^1^ Department of Medical Biotechnology, Institute of Health Sciences, Acıbadem Mehmet Ali Aydınlar University, Istanbul, Türkiye; ^2^ Health Institutes of Turkey, Istanbul, Türkiye

**Keywords:** Crimean-Congo hemorrhagic fever virus, vaccines, virus-like particle (VLP), viral vectored vaccines, DNA-based vaccines, mRNA-based vaccines

## Abstract

Crimean-Congo hemorrhagic fever (CCHF) is the most prevalent tick-borne viral disease affecting humans. The disease is life-threatening in many regions of the developing world, including Africa, Asia, the Middle East, and Southern Europe. In line with the rapidly increasing disease prevalence, various vaccine strategies are under development. Despite a large number of potential vaccine candidates, there are no approved vaccines as of yet. This paper presents a detailed comparative analysis of current efforts to develop vaccines against CCHFV, limitations associated with current efforts, and future research directions.

## 1 Background

**Figure 1:**
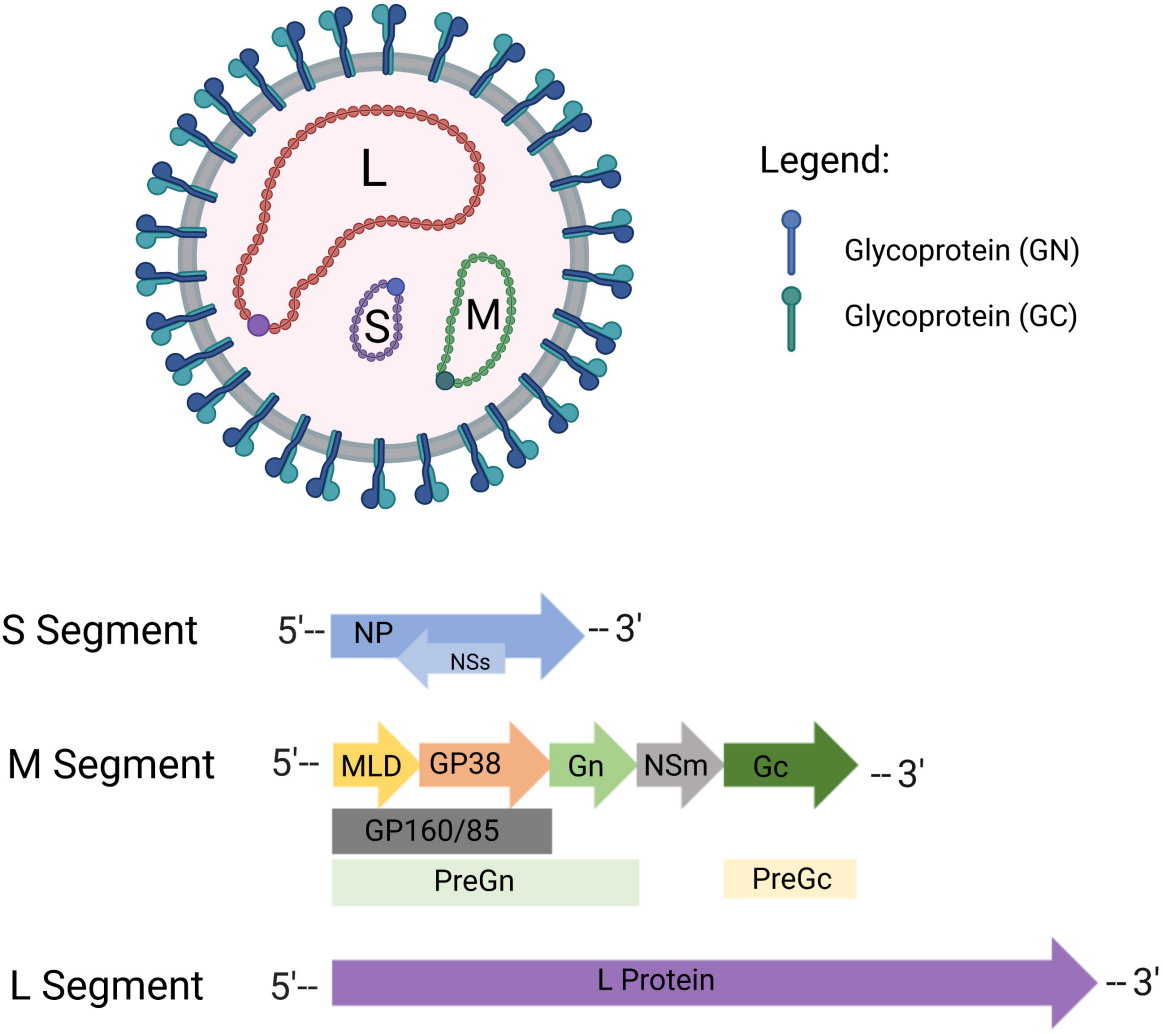
Crimean-Congo hemorrhagic fever virus (CCHFV) virion structure. CCHFV is an enveloped RNA virus with tri-segmented, negative-sense RNA. Virus envelopes contain special glycoproteins known as Gn and Gc. CCHFV consists of three genomic segments - small (S), medium (M), and large (L). In the S segment, the viral nucleoprotein (NP) is encoded in one reading frame, and the small nonstructural proteins (NSs) in an opposite-sense reading frame. The M segment encodes a glycoprotein precursor (GPC) which is processed by host proteases to produce GP160/85 domains, mucinlike domains (MLD) and GP38, as well as the medium non-structural protein (NSm). The L segment of CCHFV encoded protein contains the viral RNA-dependent RNA polymerase (RdRP). Created with BioRender.com.

The human Crimean-Congo hemorrhagic fever virus (CCHFV) is a pathogen transmitted by ticks and associated with a high mortality rate. It is a negative-sense RNA-enveloped virus belonging to the *Buny*a*virales* order, *Orthonairovirus* genus, *Nairoviridae* family. Small (S), medium (M), and large (L) segments make up the CCHFV genome which are approximately 1.6, 5.4, and 12.1 kb, respectively (**Figure 1**). Each segment contains genetic information for structural proteins involved in viral assembly, replication, encapsidation, immune escape, and transport throughout the viral life cycle. These structural proteins, namely nucleoproteins (NP), glycoprotein precursors (GPC), and RNA polymerase, are part of the polyprotein and ribonucleoprotein complex (1–3). The CCHFV genome, on the other hand, generates positive-sense viral RNA from negative-sense viral RNA using a template and translates non-structural proteins. The non-structural S protein (NSS), encoded by the S segment, is expressed at low levels and quickly degraded in CCHFV-infected cell lines *via* the proteasome, whereas the nonstructural intracellular proteins Precursor glycoprotein N-terminal (Pre-Gn), non-structural M protein (NSM), and Precursor glycoprotein C-terminal (Pre-Gc) are generated from the M segment to create a combination of partly or entirely cleaved proteins (4–6). PreGn cleavage products include non-structurally secreted proteins named GP160, GP85, and GP38 (7). Nucleoproteins and glycoproteins of CCHFV are primarily used as potential antigens in vaccine development studies. Details regarding their use as potential antigens are comprehensively discussed in the Current Limitations section.

One of the most significant tick-borne viruses, CCHFV, is endemic in areas where *Hyalomma (H. marginatum, H. anatolicum, H. truncatum, H. impeltatum, and H. impressum*) ticks are regionally dispersed, serving as both the main vector and reservoir (8, 9). This is because the virus remains present in ticks at all stages of their life cycle, from the larval stage through the nymph stage and then to maturity, and it is believed that the virus is mostly spread during the enzootic tick-vertebrate-tick cycle, which involves vertebrates such as sheep, goats, and cattle. Although CCHFV can infect several animal species, these infections are asymptomatic. The lack of animals other than humans and newborn mice intrinsically susceptible to CCHFV infection has been challenging in developing suitable animal models for CCHFV research. Under Current Limitations, a comprehensive description of animal models used in developing vaccine candidates for CCHFV is provided. Mammals can survive viremia even though ticks transmit CCHFV infection throughout their lifetimes. Humans are considered unintentional hosts of the virus and do not contribute to the transmission cycle because they are not a source of infection for ticks (10). Although CCHFV is mostly transmitted to humans through tick bites, it can also spread by direct contact with bodily fluids or tissues from infected people or animals (11). With extensive geographical distribution and re-emerging activity, CCHFV is an emerging virus with expanding geographical dispersion. It is noteworthy that Spain had two confirmed CCHFV cases in the 2010s despite the virus being predominantly observed in Eastern Europe, the Middle East, Africa, and Asia (9, 10, 12). There is a strong correlation between the expansion of *Hyalomma* species as a natural reservoir of virus and regional or local outbreaks. Environmental factors, animal dispersal, and the epidemiological history of the host play a role in the spread of these and other tick-borne viruses (11). CCHFV is included in the list of potential pathogens prioritized for public health emergencies under WHO’s R&D Plan published in 2022, along with COVID-19, Ebola virus and Marburg virus, Lassa fever virus, Middle East Respiratory Syndrome coronavirus (MERS-CoV) and Severe Acute Respiratory Syndrome (SARS-CoV) coronavirus, Nipah virus, henipavirus, Rift Valley fever, Zika virus (13).

**Figure 2:**
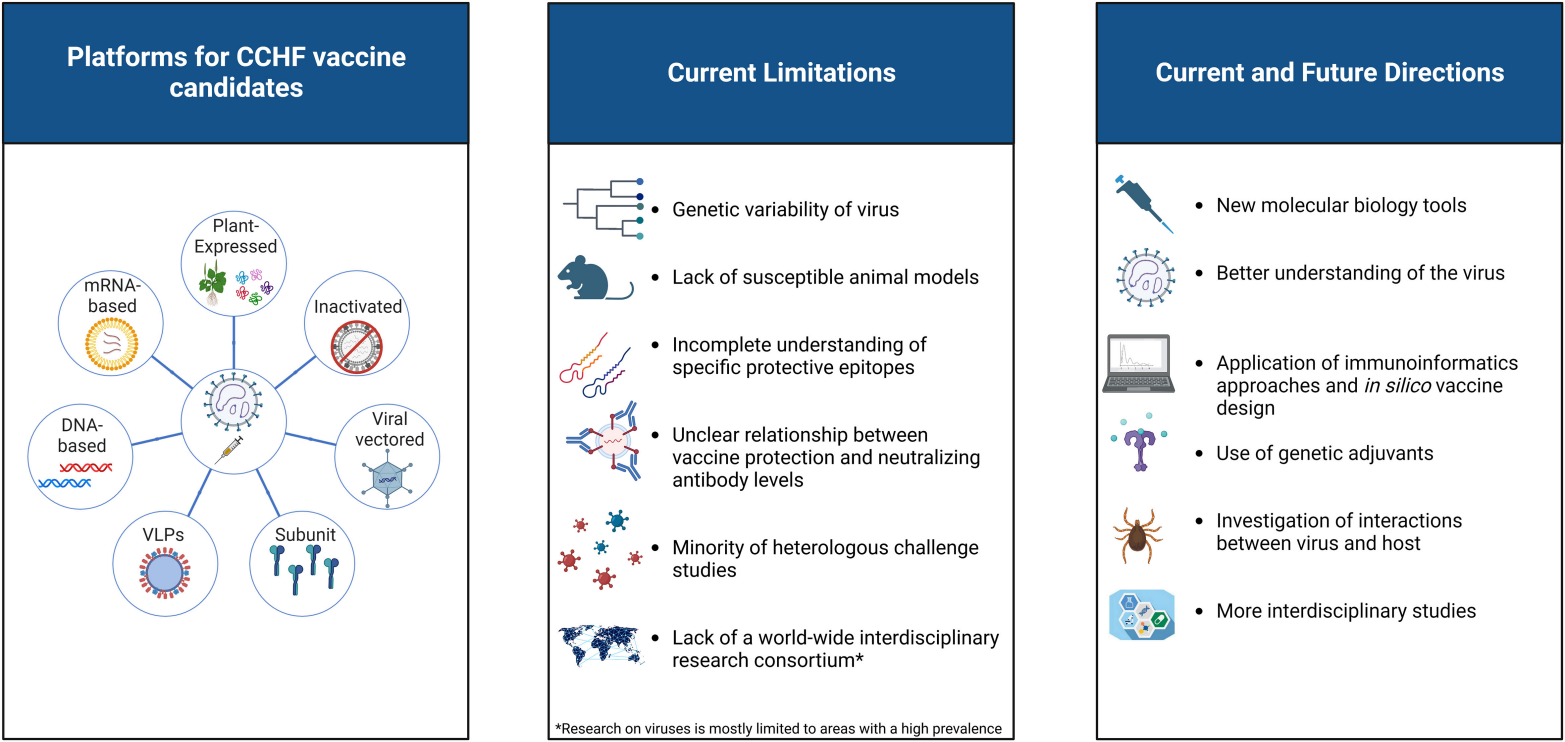
A visual representation of the current status of CCHFV vaccine platforms. The limitations of existing vaccine candidates against CCHFV, as well as several new approaches and potential strategies for vaccine development, are highlighted. Created with BioRender.com.

This review presents an in-depth and comparative analysis of different vaccine development approaches, the current status of CCHFV vaccines, their immunological properties, and limitations. Several new approaches and potential strategies for the development of a CCHFV vaccine are discussed. All these issues are summarized and illustrated in **Figure 2**.

## 2 Progress in vaccine development

A detailed overview of the outcomes of current vaccine strategies for CCHFV is presented below, including subunit, inactivated, transgenic plant-based, viral vector-based, Virus-Like Particles (VLP), and nucleic acid-based vaccines. **Table 1** presents a comparative summary of vaccine candidates.

**Table 1:** Current directions in the advancement of CCHFV vaccines.

Vaccine Types	CCHFV Antigen	Animal Model	Administration of doses	IgG Antibodies	Neutralization Antibodies	T cell response	Challenge	Protection	Reference
Plant-Expressed Vaccines	Gn and Gc (Iranian strain)	BALB/c	Feeding leaves (GnGc), 10 µg 5 doses, 1-week intervals	Yes	NT	NT	NT	NT	(14)
			Feeding roots (GnGc), 10 µg 5 doses, 1-week intervals	Yes					
			4 doses of Feeding leaves (GnGc), 10 µg 1 dose injected 5µg Gn/Gc 5 doses, 1-week intervals	Yes					
			4 doses of Feeding roots (GnGc), 10 µg 1 dose injected 5µg Gn/Gc 5 doses, 1-week intervals	Yes					
	Gn and Gc (Iranian strain) with complete/incomplete Freund’s adjuvant	BALB/c	Subcutaneously, 5 µg Gn/Gc 2 doses, 2-week intervals	Yes	NT	NT	NT	NT	(15)
			Subcutaneously, 10 µg Gn/G 2 doses, 2-week intervals	Yes					
			Subcutaneously, 15 µg Gn/Gc 2 doses, 2-week intervals	Yes					
			Subcutaneously, 20 µg Gn/Gc 2 doses, 2-week intervals	Yes					
Inactivated Vaccines	Whole Virus CCHFV Turkey-Kelkit06 strain Alum adjuvanted	IFNAR^−/−^	Intraperitoneal, 5 μg vaccine 3 doses, 3-week intervals	Yes	Yes	NT	1,000 PPFU CCHFV Turkey-Kelkit06 strain Intraperitoneally day 56	60%	(16)
			Intraperitoneal, 20 μg vaccine 3 doses, 3-week intervals	Yes	Yes			80%	
			Intraperitoneal, 40 μg vaccine 3 doses, 3-week intervals	Yes	Yes			80%	
	Whole Virus, Cell culture derived CCHFV Turkey-Kelkit06 strain Alum adjuvanted	BALB/c	Intraperitoneal, 5 μg vaccine 3 doses, 3-week intervals	Yes	Yes	NT	NT	NT	(17)
			Intraperitoneal, 10 μg vaccine 3 doses, 3-week intervals	Yes	Yes				
			Intraperitoneal, 20 μg vaccine 3 doses, 3-week intervals	Yes	Yes				
	Whole Virus, mouse brain derived CCHFV Turkey-Kelkit06 strain Alum adjuvanted	BALB/c	Intraperitoneal, 5 μg vaccine 3 doses, 3-week intervals	Yes	Yes	NT	NT	NT	
			Intraperitoneal, 10 μg vaccine 3 doses, 3-week intervals	Yes	Yes				
			Intraperitoneal, 20 μg vaccine 3 doses, 3-week intervals	Yes	Yes				
MVA Vectored Vaccines	GPC CCHF IbAr10200 strain	IFNα/β R^-/-^	Intramuscular, 10^7^ pfu MVA-GP 2 doses, 2 weeks intervals	Yes	NT	Yes	200 TCID50 CCHFV IbAr 10200 strain, Intradermal, day 28	100%	(18)
	GPC CCHF IbAr10200 strain	129Sv/Ev	Intramuscular, 10^7^ pfu MVA-GP 2 doses, 2 weeks intervals	Yes	NT	Yes	NT	NT	
MVA Vectored Vaccines	NP CCHF IbAr10200 strain	IFNα/β R^-/-^	Intramuscular,10^7^ pfu MVA-NP10200 2 doses, 2 weeks intervals	Yes	NT	Yes	200 TCID50 CCHFV IbAr 10200 strain, intradermal, day 28	0%	(19)
	NP CCHF IbAr10200 strain	129Sv/Ev	Intramuscular, 10^7^ pfu MVA-NP10200 2 doses, 2 weeks intervals	Yes	NT	Yes	NT	NT	
	NP CCHF 3010 strain	IFNα/β R^-/-^	Intramuscular, 10^7^ pfu MVA-NP3010 2 doses, 2 weeks intervals	Yes	NT	Yes	NT	NT	
	NP CCHF 3010 strain	129Sv/Ev	Intramuscular, 10^7^ pfu MVA-NP3010 2 doses, 2 weeks intervals	Yes	NT	Yes	NT	NT	
Adenovirus Vectored Vaccines	NP CCHF IbAr10200 strain	IFNAR^−/−^	Intramuscular, 1.25x10^7^ IFU Single dose	NT	NT	NT	50 TCID50 CCHFV IbAr 10200 strain, subcutaneous, day 28	1	(3)
			Intramuscular-1.25x10^7^ IFU day 0 Intranasal 10^8^ IFU day 28	Yes	NT	NT	50 TCID50 CCHFV IbAr 10200 strain, subcutaneous, day 56	78%	
	GPC CCHF IbAr10200 strain	BALB/c	Intramuscular, 5x10^7^ IU ChAd-GPC Single dose	Yes	Yes	Yes	NT	NT	(20)
			Intramuscular, 5x10^7^ IU ChAd-GPC 2 doses, 2 weeks intervals	Yes	Yes	Yes			
			Intramuscular, 5x10^7^ IU ChAd-GPC day 0 10^7^ pfu MVA-GPC day 28	Yes	Yes	Yes			
		IFNα/β R^-/-^	Intramuscular,5x10^7^ IU ChAd-GPC Single dose	Yes	Yes	Yes	200 ffu CCHFV IbAr 10200 strain, intradermal, day 28	100%	
			Intramuscular, 5x10^7^ IU ChAd-GPC 2 doses, 2 weeks intervals	Yes	Yes	Yes	200 ffu CCHFV IbAr 10200 strain, intradermal, day 28	100%	
			Intramuscular, 5x10^7^ IU ChAd-GPC day 0 10^7^ pfu MVA-GPC day 28	Yes	Yes	Yes	200 ffu CCHFV IbAr 10200 strain, intradermal, day 28	100%	
	NP CCHF Ank-2 strain	BALB/c	Intraperitoneal, 100 TCID50 Ad5-NP 2 doses, 2 weeks intervals	Yes	No	Yes	NT	NT	(21)
		IFNAR^—/—^	Intraperitoneal, 100 TCID50 Ad5-NP 2 doses, 2 weeks intervals	Yes	No	Yes	1000 TCID_50_ CCHFV Ank-2 strain, intraperitoneal, day 28	100%	
Bovine Herpesvirus Vectored Vaccines	NP CCHF Ank-2 strain	BALB/c	Intraperitoneal, 100 TCID50 BoHV4-NP 2 doses, 2 weeks intervals	Yes	No	Yes	NT	NT	
		IFNAR^—/—^	Intraperitoneal, 100 TCID_50_ BoHV4-NP 2 doses, 2 weeks intervals	Yes	No	Yes	1000 TCID_50_ CCHFV Ank-2 strain, intraperitoneal, day 28	100%	
Vesicular Stomatitis Virus Vectored Vaccines	GPC CCHF IbAr10200 strain	STAT-1^—/—^	Intraperitoneal, 10^7^ pfu VSV-GPC Single dose	Yes	Yes	NT	50 pfu CCHFV Turkey200406546 strain intraperitoneal, day 35	100%	(22)
			Intraperitoneal, 10^7^ pfu VSV-GPC 2 doses, 2 weeks intervals	Yes	Yes	NT	50 pfu CCHFV Turkey200406546 strain intraperitoneal, day 35	100%	
Subunit Vaccines	Gc-e ectodomain CCHF IbAr10200 strain (SAS adjuvanted)	STAT-1^—/—^	Intraperitoneal, 1.4 µg Gc-e 2 doses, 3 weeks intervals	Yes	Yes	NT	100 pfu CCHFV IbAr 10200 strain Intraperitoneal, day 42	0%	(23)
Subunit Vaccines	Gn ectodomain CCHF IbAr10200 strain (SAS adjuvanted)	STAT-1^—/—^	Intraperitoneal, 15µg Gn 2 doses, 3 weeks intervals	Yes	Yes	NT	100 pfu CCHFV IbAr 10200 strain subcutaneous day 42	0%	(23)
	Gc-eΔ ectodomain CCHF IbAr10200 strain (SAS adjuvanted)		Intraperitoneal, 7.5µg Gc-eΔ 2 doses, 3 weeks intervals	Yes	Yes	NT	100 pfu CCHFV IbAr 10200 strain subcutaneous day 42	0%	
	G-eGN CCHF IbAr10200 strain 201VG + Poly(I/C) adjuvanted	BALB/c	Subcutaneous, 1/5/20 µg G-eGN 4 doses, 3 weeks intervals	Yes	No	Yes	NT	NT	(24)
	G-eGC CCHF IbAr10200 strain 201VG + Poly(I/C) adjuvanted		Subcutaneous, 1/5/20 µg G-eGC 4 doses, 3 weeks intervals	Yes	Yes	Yes	NT	NT	
	G-NAb CCHF IbAr10200 strain 201VG + Poly(I/C) adjuvanted		Subcutaneous, 1/5/20 µg G-NAb 4 doses, 3 weeks intervals	Yes	No	Yes	NT	NT	
	G-eGN CCHF IbAr10200 strain		Subcutaneous, 5 µg G-eGN 4 doses, 3 weeks intervals	Yes	No	Yes	NT	NT	
	G-eGC CCHF IbAr10200 strain		Subcutaneous, 5 µg G-eGC 4 doses, 3 weeks intervals	Yes	Yes	Yes	NT	NT	
	G-NAb CCHF IbAr10200 strain		Subcutaneous, 5 µg G-NAb 4 doses, 3 weeks intervals	Yes	No	Yes	NT	NT	
Virus-like replicon particles	Gn, Gc, and NP CCHF IbAr10200 strain	IFNAR^—/—^	Intraperitoneal 10^6^ VLPs/mouse 3 doses- day 0, 28, and 49	Yes	Yes	Yes	400 FFU CCHFV IbAr 10200 strain Intraperitoneal, day 91	40%	(25)
	L -- CCHF IbAr10200 strain NP -- CCHF IbAr10200 strain GPC-- CCHF Oman-1998 strain	IFNAR^—/—^	Subcutaneous 10^5^ TCID_50_ Single dose	Yes	NT	NT	100 TCID_50_ CCHFV IbAr 10200 strain Subcutaneous, day 32	100%	(26)
		IFNAR^—/—^	Subcutaneous 103 TCID_50_ Single dose				100 TCID_50_ CCHFV IbAr 10200 strain Subcutaneous, day 32	78%	
	L -- CCHF IbAr10200 strain NP -- CCHF IbAr10200 strain GPC-- CCHF Oman-1998 strain	IFNAR^—/—^	Subcutaneous 10^5^ TCID_50_ Single dose	Yes	NT	NT	100 TCID_50_ CCHFV IbAr 10200 strain Subcutaneous, day 28	100%	(27)
DNA-based Vaccines	GPC CCHF IbAr10200 strain	BALB/c	By using gene gun 10 µg of CCHF DNA vaccine 4 doses, 4 weeks interval	Yes	Yes	NT	NT	NT	(28)
			By using gene gun 2.5 µg of each of RVFV, CCHF, HV and TBEV 4 doses, 4 weeks interval	Yes	Yes	NT	NT	NT	
DNA-based Vaccines	Gn, Gc, and NP CCHF IbAr10200 strain	IFNAR^—/—^	Intradermal 50 µg of CCHF DNA vaccine 3 doses- day 0, 28, and 49	Yes	Yes	Yes	400 FFU CCHFV IbAr 10200 strain Intraperitoneal, day 91	100%	(25)
	GPC CCHF IbAr10200 strain	IFNAR^—/—^	Intramuscular 25µg 3 doses, 3 weeks interval	Yes	Yes	NT	100 pfu CCHFV IbAr 10200 strain Intraperitoneal, day 70	71%	(29)
	GPC CCHF IbAr10200 strain	IS C57BL/6	Intramuscular 25µg 3 doses, 3 weeks interval	Yes	Yes	NT	100 pfu CCHFV IbAr 10200 strain Intraperitoneal, day 70	60%	
	GPC CCHF IbAr10200 strain	IS C57BL/6	Intramuscular 25µg, CCHFV-M10200 3 doses, 3 weeks interval	Yes	NT	Yes	100 pfu CCHFV IbAr 10200 strain Intraperitoneal, day 72	100%	(30)
	GPC CCHF IbAr10200 strain	IS C57BL/6	Intramuscular 25µg, CCHFV-M10200 3 doses, 3 weeks interval	Yes	NT	Yes	100 pfu CCHFV Afg09-2990 strain Intraperitoneal, day 72	80%	
	GPC CHFV-Afg09-2990 strain	IS C57BL/6	Intramuscular 25µg, CCHFV-M-Afg-9 3 doses, 3 weeks interval	Yes	NT	Yes	100 pfu CCHFV Afg09-2990 strain Intraperitoneal, day 72	100%	
	NP CCHF Ank-2 strain CD24 genetic adjuvanted	BALB/c	Intramuscular 50µg pV-N13 2 doses, 2 weeks interval	Yes	No	Yes	NT	NT	(31)
		IFNAR^—/—^					1000 TCID_50_ CCHFV Ank-2 strain Intraperitoneal day 28	100%	
		BALB/c	Intramuscular 40µg pV-N13 + 10µg pCD24 2 doses, 2 weeks interval	Yes	No	Yes	NT	NT	
		IFNAR^—/—^					1000 TCID_50_ CCHFV Ank-2 strain Intraperitoneal day 28	100%	
	NP + GPC CCHFV Hoti strain	Cynomolgus Macaque	Electroporation 1 mg CCHFV-NP + 1 mg CCHFV-GPC 3 doses, 3 weeks interval	Yes	No	Yes	100,000 TCID_50_ CCHFV Hoti strain intravenous day 63	NP **+** GPC improves clinical scores	(32)
	GPC CCHFV IbAr10200 LAMP1 genetic adjuvanted	HLA-A11/DR1 transgenic mice	Intramuscular 70 µg pVAX-LAMP1-CCHFV-Gc 3 doses, 3 weeks interval	Yes	Yes	Yes	100 TCID50 CCHFV tecVLPs intraperitoneal day 63	Yes	(33)
	NP CCHFV IbAr10200 LAMP1 genetic adjuvanted		Intramuscular 70 µg pVAX-LAMP1-CCHFV-NP 3 doses, 3 weeks interval	Yes	Yes	Yes	100 TCID50 CCHFV tecVLPs intraperitoneal day 63	No	
mRNA-based Vaccines	NP CCHF Ank-2 strain	C57BL/6	Intramuscular, 25 µg mRNA-NP Single dose	Yes	No	Yes	NT	NT	(34)
			Intramuscular, 25 µg mRNA-NP 2 doses, 2 weeks interval	Yes	No	Yes	NT	NT	
		IFNα/β R^-/-^	Intramuscular, 25 µg mRNA-NP Single dose	Yes	No	Yes	1000 TCID_50_ CCHFV Ank-2 strain Intraperitoneal, day 28	50%	
			Intramuscular, 25 µg mRNA-NP 2 doses, 2 weeks interval	Yes	No	Yes	1000 TCID_50_ CCHFV Ank-2 strain Intraperitoneal, day 42	100%	
mRNA-based Vaccines	Gn and Gc CCHF IbAr10200 strain	IFNAR^—/—^	Intradermal, 25 µg mRNA-NP 2 doses, 3 weeks interval	No	Yes	Yes	400 FFU CCHFV IbAr 10200 strain Intraperitoneal, day 56	100%	(35)
	NP CCHF IbAr10200 strain		Intradermal, 25 µg mRNA-NP 2 doses, 3 weeks interval	Yes	No	Yes	400 FFU CCHFV IbAr 10200 strain Intraperitoneal, day 56	100%	
	Gn, Gc, + NP CCHF IbAr10200 strain		Intradermal, 25 µg mRNA-NP 2 doses, 3 weeks interval	Yes	Yes	Yes	400 FFU CCHFV IbAr 10200 strain Intraperitoneal, day 56	100%	
	NP CCHFV Hoti strain	C57BL6/J	Intramuscular, 2.5 µg rep-NP 2 doses, 4 weeks interval	Yes	No	No	100 TCID50 CCHFV UG3010 strain Intraperitoneal, day 56	100%	(36)
	GPC CCHFV Hoti strain		Intramuscular, 2.5 µg rep- GPC 2 doses, 4 weeks interval	No	No	Yes	100 TCID50 CCHFV UG3010 strain Intraperitoneal, day 56	No	
	NP+GPC CCHFV Hoti strain		Intramuscular, 5 µg rep-NP+rep-GPC 2 doses, 4 weeks interval	Yes	Yes	No	100 TCID50 CCHFV UG3010 strain Intraperitoneal, day 56	100%	

### 2.1 Plant-expressed vaccines

Genetically engineered plants have been an integrated platform for biopharmaceutical production for the past 30 years. Both transiently and stably transformed plants with chloroplast expression systems are now used as expression techniques to utilize plant cells as host cells to produce recombinant proteins. The main benefits of plant-based recombinant production platforms include the lack of human pathogen replication (which minimizes the chance of contamination and reduces the purification process), use of simple bioreactors, efficient synthesis of complex proteins (multimeric or glycosylated), and capacity to produce large quantities of biologically active proteins (37).

In the study by Ghialsi et al.; Gn and Gc glycoproteins from CCHFV were expressed in transgenic plants and administered to mice *via* oral route to evaluate their oral immunogenicity. Gn and Gc glycoproteins from the M segment of the Iranian strain were codon-optimized for plants, genetically engineered into one reading frame, and cloned into a plant-cloning vector. The expression of Gn/Gc glycoprotein (GenBank accession number: HM537014) was determined in the hairy roots and leaves of the transgenic plants and then analyzed by Western blotting and specific Enzyme-Linked ImmunoSorbent Assay (ELISA). Six groups were formed for the immunization studies. Of these, the first two groups were fed only transgenic leaves or roots, whereas the other two groups were fed transgenic leaves or roots and injected subcutaneously with the plant-made CCHFV glycoprotein (fed/boosted). The positive control group was vaccinated with an attenuated CCHFV vaccine, whereas the negative control group received no immunization (14). This study demonstrated the feasibility of immunizing animals with edible materials derived from genetically modified plants, particularly in the context of CCHFV. This study, however, did not evaluate neutralizing antibodies, conduct challenge studies, or conduct efficacy experiments in an animal model of CCHFV. Notably, the transgenic plant vaccine showed the possibility of including only the Gn and Gc portions of the CCHFV M segment as an edible vaccine (38). In line with this study, the same research team engineered a novel plant-optimized gene cassette comprising the Gn/Gc genes. Bioinformatic tools were used to assess parameters such as surface accessibility, antigenicity, and N-glycosylation sites of Gn and Gc glycoproteins. Tobacco plants were then genetically modified using transient and stable transformation methods. Mice were immunized with the obtained Gn/Gc glycoproteins at four different doses (5, 10, 15, or 20 μg). The positive control group was immunized with a CCHFV vaccine consisting of inactivated viral particles (15). Although the study publication mentioned that plant-derived Gn/Gc protein elicited high levels of anti-CCHFV glycoprotein IgG antibodies in mice, details of immunological studies of these findings are not provided.

### 2.2 Inactivated vaccines

Inactivated vaccines are produced by cultivating infectious agents in the laboratory and rendering them non-pathogenic through chemical or physical processes. Vaccines such as polio (39), influenza (40), and rabies (41) have been commercially available and licensed for many years, employing a well-established and effective vaccination approach. While research continues to develop alternative long-term strategies tailored to new pathogens, especially during pandemics, regulatory authorities support the emergency use of inactivated vaccines due to their well-established history and well-defined production processes (42). However, inactivated vaccines may generate a lower adaptive immune response than other vaccine types, necessitating more frequent booster dose administration. Additionally, since the level of protection can vary against different strains or variants of the pathogen, periodic vaccine updates may be required (43).

Although a globally recognized vaccine for CCHFV is not currently available, an inactivated vaccine, referred to as the Bulgarian vaccine from the National Center for Infectious and Parasitic Diseases (BulBio-NCIPD Ltd.), has been utilized in Bulgaria since 1974, albeit without approval from the Food and Drug Administration (FDA) and the European Medicines Agency (44). The Bulgarian vaccine is derived from CCHFV-infected suckling mice brain tissue, which is inactivated through heating at 58°C and treatment with chloroform. It is administered subcutaneously, requiring multiple booster doses in individuals aged ≥ 16 years who belong to high-risk groups (45). Research has indicated that individuals vaccinated with the inactivated Bulgarian vaccine exhibit robust T-cell activity against CCHFV. In spite of the fact that research suggests individuals vaccinated with the inactivated vaccine have robust T-cell activity against CCHFV, experimental groups receiving multiple doses (four doses) demonstrated significantly higher levels of interferon-secreting effector T cells and IgG antibody responses than those receiving only one dose. Despite booster vaccinations, neutralizing antibody levels in vaccinated individuals are insufficient (46). Contrary to these experimental findings, in Bulgaria, there was a notable decline in the number of reported CCHFV cases from 1105 to 279 between 1974 and 1996, with fewer than 20 cases reported annually after 1996. However, it should be noted that this decline might be attributed to changes in the epidemiology and ecology of CCHFV, independent of the vaccine’s efficacy, as well as behavioral changes resulting from increased awareness of CCHFV and reduced tick exposure (47). Though utilized within the country, the Bulgarian vaccine is unsuitable for widespread global application mainly due to safety issues and the absence of efficacy trials. Safety concerns are based on the possibility of contamination with mouse neural tissue due to the vaccine’s origin, the possibility of triggering autoimmune and allergic reactions. Moreover, the vaccine necessitates high-containment facilities, like Biosafety Level-4, further limiting its suitability for broader usage worldwide (48). Additionally, reimmunization every five years is necessary to maintain immunity. Individuals under the age of 16 years are not eligible for vaccination, leaving a portion of the population susceptible to the virus. Furthermore, the vaccine has not been experimentally proven to be protective against virus challenges in mouse studies. The effectiveness of the Bulgarian vaccine has not yet been established in controlled clinical trials.

Various attempts have been made to develop inactivated vaccines to counteract the regulatory challenges associated with mouse brain-derived Bulgarian vaccine (16, 17). In the study published in 2015, the CCHFV Turkey-Kelkit06 strain was cultured in Vero-E6 cells, purified, and then inactivated using formalin to develop a CCHFV preparation. The vaccine was formulated with Alum as an adjuvant and administered intraperitoneally to type I interferon receptor-deficient (IFNAR^−/−^) mice with three different doses (5, 20, and 40 µg), utilizing a prime, boost, and boost strategy. Immunogenicity studies revealed that the group receiving the 5 µg dose showed the lowest levels of neutralizing antibody titers *in vitro*, and the increase in antibody titer was dose-dependent. Despite variations in neutralizing antibody levels, similar survival rates (80%) were observed in the 20 µg and 40 µg dose groups of IFNAR^−/−^ mice when subjected to a lethal challenge infection (16). This study demonstrated that the cell culture-based vaccine could indeed induce neutralizing antibodies.

A study conducted in 2021 utilized both suckling mice and Vero-E6 cells to cultivate the Kelkit06 CCHFV virus strain. The vaccine candidates were then purified using a sucrose gradient and inactivated with formalin. Six groups of BALB/c mice were administered intraperitoneal route doses of 5, 10, and 20 μg of two types of vaccine candidates: one derived from cell culture and the other derived from mouse brains. Both vaccine candidates were formulated with an alum adjuvant. The mice in each group received the same doses of the formulated vaccine and vaccine type for the second and third doses, with a 3-week interval between each dose. A seventh group of mice served as a negative control and received a Phosphate Buffered Saline-mock vaccine with alum adjuvant. The study found that the vaccine candidate derived from cell culture significantly increased IgG titers and elicited a broad humoral immune response with neutralizing antibodies in BALB/c mice. Comparative analysis until up to 1 year after immunization revealed that the cell culture-derived vaccine candidate produced significantly higher levels of neutralizing antibodies than the mouse brain-derived vaccine (17). Consequently, it was determined that the cell culture-derived vaccine candidate was capable of triggering a potentially more robust protective response than the vaccine candidate derived from the mouse brain.

### 2.3 Viral vectored vaccines

The basis of viral vector vaccines depends on the modified viral particles that contain one or more foreign genes encoding antigens of the target pathogen, and which have been used for over 40 years (49, 50) Compared with traditional vaccines, viral vector vaccines provide several benefits. They can be assumed to be harmless because they do not contain the whole virus and trigger both innate and adaptive immune responses. Due to the expression of numerous pathogen-associated molecular models and the activation of the innate immune system, viral vectors have natural adjuvant characteristics. Viral vectors can be designed to transport antigens to specific cells or tissues, thereby increasing their safety and lowering reactogenicity (51). They can also be replication-competent or replication-deficient (52). Numerous preventive vaccinations based on viral vectors have started Phase III clinical studies or have already been approved (53). The major limitation of viral vector vaccines is the possibility of immune protection against the antigenic components of the vector, either already existing or developing, weakening the immune response to the targeted gene. Potential solutions include using heterologous prime-boost vaccination regimens and delivering of higher vaccine doses if tolerated. These strategies aim to maximize the immune response to the target transgene and improve vaccine efficacy while reducing interference from immune responses related to the vector (51).

Due to its capacity to stimulate humoral and cellular immunity and to support high-level expression of recombinant genes both *in vitro* and *in vivo* with post-translational modifications in the host cell, the Modified Vaccinia virus Ankara (MVA) from the poxvirus family is frequently used as a carrier vector in viral vector vaccines (54). In the context of CCHFV, MVA was employed as the carrier vector for the first viral vector vaccine, enabling the expression of the complete glycoprotein derived from the CCHFV M segment. Two strains of mice, including three types of interferon receptor knockout mice (IFNARα/β/γR^−/−^) susceptible to CCHFV disease, were administered intramuscularly in an animal study, and challenge studies were carried out intradermally to simulate natural tick bites. Extensive cellular and humoral immunity against CCHFV had been demonstrated by the end of the studies, allowing protection against severe disease without any apparent symptoms. Even though the animals that survived the challenge did not exhibit any clinical symptoms, histology, and viral load studies revealed that they had been exposed to the virus (18). Although it has been shown that prime-boost vaccination with MVA-GP produces both humoral and cellular immunity, in a study carried out to determine which immune arm is more effective in the protective effect, animals that received both serum and CD3+ T lymphocytes from vaccinated mice were compared with animals that received serum or CD3+ T lymphocytes. A substantial improvement in the time to death was observed. However, it has been observed that CD3+ T lymphocytes cannot provide protective effects on their own (55). In contrast to the glycoprotein from the M segment, no protection was observed in the CCHFV virus challenge study. However, antigen-specific immunogenicity was observed in mice when the study was repeated with NP from the S segment (19).

Due to their high transduction efficiencies, relatively large capacities for transgenes, and other beneficial characteristics such as high titers, adenovirus vectors (Ad-vector) are frequently utilized in gene transfer investigations. Additionally, the Ad-vector contains components that are recognized by a variety of PRRs, such as toll-like receptors, retinoic acid-inducible gene-I -like receptors, and cyclic guanine adenine synthases, which function as adjuvants to slightly activate innate immunity (56). However, it does not cause strong innate immune reactions, such as cytokine storms, or significant harm to transduced cells. These factors make it possible to activate adaptive immunity to transgenic products using Ad-vectors without causing negative side effects (57). The vaccine study published in 2018 focused on expressing the CCHFV NP using the human adenovirus type 5 vector. In this study, immune responses against the CCHFV challenge in mice vaccinated with Ad-N were measured to examine the vaccine’s protective effects. The results demonstrated that IFNAR^−/−^ mice immunized with Ad-N generated IgG responses targeting the CCHFV NP and exhibited partial protection against the CCHFV challenge. Notably, a single dose of Ad-N provided 30% protection in IFNAR^−/−^ mice, whereas a prime-boost regimen increased the protection to up to 78% against the CCHFV challenge (3). Recently, a vaccine against CCHFV was created using replication-deficient chimpanzee adenovirus viral vector technology, which has attracted considerable attention during the SARS-CoV-2 pandemic. In this study, the immunogenicity and protective effectiveness of the CCHFV GPC -expressing adenoviral vector vaccine (ChAdOx2 CCHFV) either individually or in combination with the MVA CCHFV vaccine (18) were assessed against CCHFV. Strong antibody responses and interferon-gamma (IFN-γ)-mediated cellular immunity emerged following the vaccination of immunocompetent BALB/c and immunodeficient A129 mice (lack of interferon-alpha/beta receptor 2 (IFNAR2) gene) with various combinations of these vaccine candidates. Complete protection against CCHFV was attained in the A129 lethal mouse model in which ChAdOx2 CCHFV was administered as a single dose or following homologous or heterologous prime-boost immunization regimens with the MVA CCHFV vaccine. However, heterologous prime-boost immunization provided a high level of protection compared with other immunization regimens in the study (20).

Bovine herpesvirus 4 (BoHV-4) is a viral vector with a high potential for vaccination studies due to several factors, such as its straightforward genomic structure, simplicity of genome manipulation, lack of significant pathogenicity in humans, lack of potential for transformation in infected cells, and lack of vector-neutralizing antibodies in humans (58). The BoHV-4-based viral vector (BoHV4-DTK-CCHFV-N) vaccine was created for the first time in research conducted by Farzani et al. in an effort to create a vaccination strategy against the CCHFV NP. Adenovirus type 5 and DNA vector-based vaccines commonly utilized for CCHFV protection were compared to determine the immunogenicity of the vaccine candidate in BALB/c mice and the protection potential in IFNARα/β/γR^−/−^ animal models. In both mouse models, vaccination significantly increased cytokine levels as well as the production of specific antibodies. Only the BoHV4-DTK-CCHFV-N and Ad5-N vaccine designs, completely protected against the CCHFV Ank-2 strain and significantly increased levels of IL(Interleukin)-6 and Tumor necrosis factor-alpha (TNF-α) in IFNARα/β/γR^−/−^ animals after the challenge trials (21).

Recombinant vesicular stomatitis virus (rVSV) has been utilized as a vaccination platform to express foreign antigens for numerous viral infections in recent years (59). The target pathogen glycoprotein was frequently inserted into the region where the VSV envelope glycoprotein was truncated in the vaccine design. By accomplishing this replacement, the pathogenicity of VSV is diminished, and vaccine safety is enhanced (60). The CCHFV GPC was expressed utilizing rVSV for the first time in the research published in 2019 to develop a viral vector-based vaccine. Following intraperitoneal vaccination of Signal Transducer and Activator of Transcription 1 deficient mice (STAT1^−/−^) with a single dose of rVSV-GP, the STAT1^−/−^mice were challenged with a clinical strain of CCHFV. The rVSV-GP vaccine candidate offered complete protection, and anti-CCHFV-GP IgG titers and neutralizing antibodies were detected in the survived mice (22).

### 2.4 Subunit vaccines

Subunit vaccines are composed of specific antigenic components of a pathogenic organism that can stimulate a protective immune response (61). These vaccines can be created using recombinant DNA technologies. In the case of CCHFV, subunit vaccines are primarily produced by utilizing the baculovirus-insect cell expression system. Baculoviruses offer several advantages for producing viral and parasitic antigens, such as their ability to accommodate large DNA fragments of up to 38 kb, their non-integration into the host cell genome, and their capacity for post-translational modifications. The use of the baculovirus expression system (BEVS) originated in the 1980s when the promoter of the polyhedrin protein, a major structural protein of the virus, was discovered (62). The production process with BEVS consists of the steps to obtain the recombinant virus by cloning the antigenic gene region into baculovirus, transfecting insect cells with this recombinant baculovirus, propagating the baculovirus in insect cell culture, and obtaining it by purification of the protein produced during insect culture, as in the production of a standard monoclonal antibody (63).

Viral antigens are typically complex proteins with high biological activity and require post-translational modifications such as glycosylation, disulfide bond formation, myristoylation, and phosphorylation. Insect cells play a crucial role in the development of viral vaccines as they possess the ability to perform these modifications (64). CERVARIX^®^, a vaccine based on virus-like particles (VLPs) developed by GSK-Rixensart in Belgium, targeting Human Papilloma Virus (HPV), and Flublok, a recombinant protein influenza vaccine developed by Protein Sciences Corporation in Meriden, CT, USA, are prominent examples of vaccines produced in insect cells and approved by the FDA (65).

The first attempt to develop a subunit vaccine against CCHFV, using an insect expression system was published in 2015 by Kortekas et al. In this study, the ectodomains of structural glycoproteins Gn and Gc of the CCHFV IbAr10200 strain were produced using a Drosophila insect cell-based expression system. Gn or Gc ectodomains formulated with adjuvant after purification were used for IP route vaccination of STAT1^−/−^ mice with prime-boost strategy. Although a neutralizing antibody response was obtained after the booster dose, the challenge study showed that STAT1^−/−^ mice were not protected against the CCHFV infection challenge (23).

In a recent study on subunit vaccine development against CCHFV, the GEM-PA surface display system was used to express the ectodomains (eGN, eGC, and NAb) of the structural glycoproteins of CCHFV. The GEM-PA surface display system uses non-viable and non-genetically modified gram-positive bacterial cell and gram-positive enhancer matrix (GEM) particles. These particles serve as platforms for binding external proteins of interest using a high-affinity binding domain. The binding domain, known as the protein anchor (PA), is derived from a peptidoglycan hydrolase called AcmA, found in *Lactococcus lactis*. In essence, the GEM-PA system uses GEM particles as a base and the PA domain of AcmA to facilitate the attachment of foreign proteins to the surface of these particles (66, 67). In this study, the GEM-PA surface imaging system was used to provide a flexible and versatile purification strategy for the CCHFV subunit antigens. BALB/c mice were given vaccine candidates formulated with Montanide ISA 201VG plus Poly (I: C) adjuvant through three subcutaneous injections. These vaccines successfully elicited both GP-specific humoral and cellular immune responses in mice.

Furthermore, all three vaccine candidates demonstrated elevated levels of TNF-α, IL-6, and IL-10 cytokines in the supernatant of stimulated splenocytes *in vitro*. However, only the eGC vaccination group exhibited the presence of neutralizing antibodies, with the highest neutralizing titer. This finding suggests that the G-eGC vaccine may induce a more potent humoral immune response (24). Notably, this study marks the first utilization of GP’s highly conserved and neutralizing antibody region among vaccine candidates developed against CCHFV

### 2.5 Virus-like replicon particles

Virus-like particles (VLPs) are icosahedral or rod-shaped nanoscale entities that form assembled viral structural proteins resembling the size and shape of authentic viruses. These VLPs lack viral genetic material. Viral constructions can be formed and reconstructed once the viral structural proteins are translated and self-assembled *in vivo* or *in vitro* (68, 69). Compared to other subunit vaccines, VLPs have many inherent advantages. They resemble pathogen-associated structural patterns that can be quickly recognized by immune system cells and molecules. This causes cell recruitment and immune processing pathways to be connected to their parent viruses because of their morphological similarity to those parent viruses, which have a highly repetitive immunogenic surface structure. They are not pathogenic since they lack an entire virus genome and are unable to replicate or infect the host. By doing this, they vastly improve the safety profile compared to live attenuated vaccinations (70, 71). Owing to all these benefits, numerous commercial vaccines such as Recombivax HB^®^ (Merck & Co) Engerix-B^®^ (GlaxoSmithKline) and against Hepatitis B virus, Gardasil^®^ (Merck & Co) and Cervarix^®^ (GlaxoSmithKline) against HPV have acquired regulatory health authority approval and numerous vaccines are still being developed in clinical research (72).

Despite all these benefits, there are several unresolved concerns regarding the use of VLP-based vaccines. First, manufacturing techniques can be more complicated and technically demanding than conventional vaccine manufacturing techniques. Second, because they lack a viral genome, VLPs are susceptible to instability by modifications implemented in the production and purification procedures, even though they are multimeric structures that are typically more stable than subunit vaccines. Finally, since these vaccines utilize novel technologies compared to conventional vaccines, regulatory approval procedures and relevant guidelines may be different. Further analyses and evaluations are required to verify the safety and effectiveness of the vaccines. Despite these additional challenges, the use of VLP-based vaccines is increasing (73, 74).

The first attempt to create a VLP vaccine for CCHFV was led by Devignot et al. in 2015. Reverse genetics has been used to produce transcriptionally competent virus-like particles (tc-VLPs) that display CCHFV Gn and Gc proteins on their surface, contain a reporter minigenome containing the L protein, and are encapsulated by NP (75). This study paved the path to further CCHFV vaccine and antiviral development studies by enabling CCHFV infection studies under Biosafety Level-2 conditions, as CCHFV needs Biosafety Level -4 conditions (29, 30, 76, 77). In a subsequent study, mice were administered three intraperitoneal doses of both the novel DNA-based vaccine and the tc-VLPs displaying the structural proteins of strain CCHFV IbAr10200. However, tc-VLPs protected only 40% of the mice in the IFNAR^−/−^ mouse model, despite the production of significant neutralizing antibody titers (25).

In another vaccine candidate development study against CCHFV, a virus-like replicon particle (VRP) which contains the full-length S and L genome segments, but not the entire M segment, but only the GPC coding region, was used to limit replication to a single cycle. Afterward, it was investigated whether IFNAR^−/−^ mice were protected against CCHFV challenge by a single dose of VRP vaccination. Subcutaneous vaccination was administered at either a high (10^5^ 50% Tissue Culture Infectious Dose (TCID_50_)) or low (10^3^ TCID_50_) dose. Only 5/10 of the low-dosage vaccinated mice developed anti-NP antibodies, and 9/10 developed anti-Gc antibodies, whereas mice given a high dose of VRP developed significant levels of IgG antibodies to both NP and Gc. All mice in the negative control group and mice that were given low doses of VRP after the challenge exhibited clinical symptoms of CCHFV disease. However, mice that received a high dose of VRP vaccination, did not show any clinical symptoms (26). These results demonstrate that a single dose of this VRP vaccine candidate is safe and effective in immunosuppressed IFNAR^−/−^ mice. To further assess the future application potential of the VRP vaccine, its protective effect against different CCHFV strains was evaluated. In the challenge study, the CCHFV IbAr 10200, CCHFV-Turkey, and CCHFVOman-97 strains were injected subcutaneously. The results of the study demonstrated that IFNAR^−/−^ mice were heterologously protected from disease following a single dose of VRP vaccination (27).

### 2.6 DNA vaccines

The use of DNA-based vaccination as a novel strategy in vaccine development began in the late 1990s (78). Its basic functional concept is based on the administration of plasmids, which contain the gene region required for the expression of particular viral antigens or proteins in muscle or skin cells, often under the control of the eukaryotic promoter (79, 80). Antigens co-expressed with the plasmid are presented on the surface of the cells after entering the cell, inducing both humoral and cellular immune responses (81). The capacity to induce direct antigen production in cells by simulating viral infection, ease of manufacture, and high stability are significant benefits of DNA-based vaccinations. Nevertheless, similar to any emerging technology, DNA-based vaccines have certain constraints. These include challenges associated with delivering the vaccines to cells, the theoretical possibility of integration into the host genome, and the requirement for multiple doses due to their relatively low immunogenicity compared to traditional vaccines (82, 83). However, ongoing global research is dedicated to addressing these limitations and enhancing DNA vaccine safety and effectiveness. The recent Emergency Use Authorization granted to ZyCoV-D, a DNA-based COVID-19 vaccine, by the regulatory agency in India marks a significant milestone in the advancement of DNA-based vaccine technologies (84).

The first DNA-based vaccine against CCHFV was designed in 2006 to express the GPC of the IbAr 10200 CCHFV strain. The study involved administering the CCHFV DNA-based vaccine construct three times, alone or in combination with other DNA-based vaccine constructs for Hantaan, Rift Valley fever viruses encoding GPC, and encephalitis viruses encoding premembrane and envelope genes. Only 50 percent of the vaccinated BALB/c mice developed neutralizing antibodies *in vitro*, either in combination with the CCHFV DNA vaccine or other vaccine constructs (28). However, the induction of cell-mediated immune responses was not evaluated, and challenge studies could not be performed to evaluate the vaccine candidate confers protective immunity, as there was no animal model for CCHFV at the time of the study.

Developing the IFNAR^−/−^ knockout mouse model mimicking human CCHFV pathogenesis, has expedited vaccine development research. In a 2017 publication, the immunogenicity and protective efficacy of two novel CCHFV vaccine candidates were examined: a tc-VLPs -based vaccine and a DNA-based vaccine that encodes a ubiquitin-linked version of the Gc, Gn, and N proteins of CCHFV. The DNA vaccine demonstrated robust and 100% preventive immunity against lethal CCHFV challenges. In contrast to previous studies, this research revealed significant increases in Th1-type biomarkers (IFN-γ, TNF-α, IL-12 p70, and IL-2) in both DNA-immunized and DNA-VLP-immunized IFNAR^−/−^ mice (25). These findings highlight the importance of a Th1-type immune response for adequate protection against CCHFV.

Furthermore, the presence of high levels of neutralizing antibodies in surviving mice suggests the need for further investigations in future studies. Having demonstrated that vaccination with a DNA-based vaccine containing NP, Gn, and Gc proteins of the CCHFV IbAr 10200 strain provided protection in homologous lethal challenge experiments, further experimental data were required. The studies above were made possible by the development of Cynomolgus macaques as a non-human primate model based on the CCHFV Hoti strain (85). In research from 2020, two ubiquitin-antigen fusion plasmids encoding the NP and GPC proteins of the CCHFV Hoti strain were administered to Cynomolgus macaques *via* electroporation three times, three weeks apart. Twenty-seven days after the last immunization, the CCHFV Hoti strain was administered to all the animal groups as a challenge study. In the non-human primate model, the study showed that the DNA-based vaccine produced an immune response that provided CCHFV protection. A challenge study performed on vaccinated animals revealed CCHFV-specific antibodies and T-cell responses; however, viremia, viral shedding, and viral load were dramatically reduced in vital tissues, including the liver, after the challenge study (32). These results imply that the vaccine might proceed to human clinical trials. The impact of administering the same DNA vaccine constructs in combination with the prime-boost regimen against CCHFV was examined after it was demonstrated that DNA-NP and DNA-GPC vaccines administered with the prime-boost-boost regimen in cynomolgus macaques provided significant protection against viral replication and disease. This combined DNA-NP and DNA-GPC vaccine has been shown to offer more protection against CCHFV infection than a triple DNA-NP or DNA-GPC vaccination given individually (86).

In another study published in 2017, a DNA vaccine was created to express the M-segment GPC gene of CCHFV. The immunogenicity and protective efficacy of the vaccine were assessed by administering it through muscle electroporation in two different mouse disease models: IFNAR^−/−^ mice and a newly developed transiently immunosuppressed mouse model. Following triple vaccination with 25 μg, robust antigen-specific humoral immune responses were observed in both mouse models, with neutralizing titers detected. When challenged with CCHFV, the DNA-based vaccine provided protection to over 60% of the animals, preventing lethal disease (29). The majority of vaccine studies for CCHFV involve homologous challenge tests using laboratory-adapted strains, which have certain limitations. To overcome these limitations, the same research team conducted further investigations in their 2017 study (CCHF-M10200) by evaluating immune response enhancement through increased vaccine dosage in homologous and heterologous challenge tests in mice. They tested a 50 μg dose of the CCHF-M10200 vaccine and developed a vaccine (CCHFV-MAfg09) based on the M-segment of the clinically relevant CCHFV-Afg09-2990 strain. The results showed that a 50 μg dose of the CCHF-M10200 vaccine provided complete protection in the homologous challenge study and 80% protection against the heterologous CCHFV-Afg09-2990 strain. Similarly, a newly designed DNA vaccine (CCHFV-MAfg09) demonstrated full protection against the CCHFV-Afg09-2990 strain in a homologous challenge study. However, no direct correlation was observed between anti-CCHFV-GPC-IgG titers or anti-GPC-specific T cell populations and survival rates in heterologous challenge tests. In the second phase of the study, a DNA vaccine (ΔMLDΔGP38) was developed to investigate the immunogenicity of the CCHFV vaccine by expressing an M-segment of CCHFV-IbAr 10200 with a deletion of the Mucin-Like-Domain (MLD) and GP38 regions. The ΔMLDΔGP38 vaccine served as a negative control in terms of immunogenicity (69). However, previous studies have shown that treatment with an anti-GP38 monoclonal antibody can prevent liver damage in mouse models (76), suggesting that GP38 may be a potential target protein for vaccines, which requires further investigation. In 2019, a DNA vector was designed to express the nucleocapsid protein (N) of CCHFV, and the CD24 protein was utilized as a potential adjuvant. CD24 was chosen for its ability to stimulate B and T cells, ensuring adequate antibody production against N protein. The capacity of vaccine candidates to induce cytokine responses, as well as total and specific antibody production, was evaluated in BALB/c mice, and a challenge experiment was conducted using IFNAR^−/−^ mice. The results demonstrated that the N-expressing DNA construct, whether administered alone or in combination with the pCD24 vector, generated significant cellular and humoral responses in BALB/c mice, albeit with variations in certain cytokines and total antibodies. However, the antibodies produced did not possess neutralizing properties in a virus-neutralization assay. Analysis of cytokines in the IFNAR^−/−^ mouse model showed elevated levels of IL-6 and TNF- α, whereas the challenge study revealed the protective potential of the N-expressing construct (31). In summary, targeting the S segment has proven to be a practical approach for developing vaccines against CCHFV. In a Chinese study published in 2023, three separate DNA-based vaccines were created by fusing the CCHFV IbAr10200 strain’s nucleocapsid protein (NP), glycoprotein N-terminal (Gn), and C-terminal (Gc) proteins individually with Lysosome-associated membrane protein 1 (LAMP1), and a transgenic mouse model was used to assess vaccine immunogenicity and protective effectiveness. CCHFV transcription and entry-competent virus-like particles (tecVLPs) were employed in the present study instead of the intact virus in the challenge experiments, and HLA-A11/DR1 transgenic mice were used in the animal studies, in contrast to previous studies on CCHFV vaccine development. The human HLA-A11 (MHC-I) and HLA-DR1 (MHC-II) genes were introduced into the wild-type C57BL/6 mouse genome to create transgenic mice, which are theoretically expected to mimic the antigen presentation pathway in humans partially. Three doses of LAMP1-CCHFV-NP vaccination in mice resulted in a balanced Th1 and Th2 response, specific anti-NP antibodies, and CTL responses as well as the strongest protection against CCHFV tecVLP infection. As opposed to LAMP1-CCHFV-NP, mice immunized with LAMP1-CCHFV-Gc exhibited mostly specific anti-Gc and neutralizing antibodies as well as some protection against CCHFV tecVLPs infection. LAMP1-CCHFV-Gn-vaccinated mice exhibited only specific anti-Gn antibodies and did not offer sufficient defense against CCHFV tecVLPs infection. According to these findings, LAMP1-CCHFV-NP may be a practical and efficient vaccine candidate for CCHFV (33).

### 2.7 mRNA vaccines

To stimulate an immune response against a specific pathogen, mRNA vaccines include components that facilitate the translation of the target pathogen’s antigen. *In vitro* transcribed (IVT) mRNA is expected to have the following elements to resemble native mRNA: a 5’ cap, a 5’ untranslated region (UTR), an open reading frame (ORF) encoding the antigen, a 3’ UTR, and a poly(A) tail (87, 88). IVT mRNA cannot cross the anionic lipid bilayer of cell membranes since it is a structurally large and negatively charged molecule. In order to enable the transport of mRNA across the cell membrane and prevent it from being absorbed by cells of the human innate immune system and being destroyed by nucleases, carrier systems such as lipid nanoparticles (LNPs) are required (89). Although mRNA vaccines have been used for therapeutic purposes for a long time, the approval of the two-mRNA vaccine candidates against Covid-19, developed by BioNtech/Pfizer and Moderna Inc, by international health authorities during the SARS-CoV-2 pandemic has heightened the interest in mRNA vaccines for preventative usage against viral diseases.

Research on using mRNA-based constructs as a novel expression platform against viral infections existed even before the Covid-19 mRNA vaccines. The S segment of the CCHFV Ank-2 strain was inserted into a conventional mRNA structure in research by Farzani et al. published in 2019 without optimization. mRNA was delivered directly without the use of a carrier mechanism, unlike mRNA vaccines that are now available. This experimental, first-of-its-kind mRNA-S segment structure has been evaluated for immunogenicity and protection against viral challenge in 2 distinct mouse models: C57BL/6 and IFNARα/β/γR^−/−^. IFN-γ cytokines and anti-nucleocapsid IgG1 and IgG2a antibodies have been reported to be significantly produced by mRNA vaccination in both single and booster dose groups (34).

As a result of novel mRNA vaccine technologies, two alternative mRNA-LNP constructs were created in 2022 using glycoproteins (Gc and Gn) or NP of the CCHFV IbAr10200 strain. Immunocompetent and immunocompromised mice received individual and combined administrations of vaccine candidates as part of the immunization research. A lethal dose of CCHFV IbAr10200 was administered to all animals five weeks following the second inoculation. IFNAR^−/−^ mice that received the vaccination appeared to survive the challenge and were able to manage the infection, but they did not develop sterile immunity. After all vaccination and challenge experiments, it was shown that vaccines effectively induced robust humoral and cellular immunity, while offering 100% protection against CCHFV infection (35). Leventhal et al. (36) used the Venezuelan Equine Encephalitis Virus and a cationic nanocarrier delivery method to create a self-replicating RNA vaccine for the CCHFV NP and GPC. A heterologous challenge investigation on immunized mice revealed that the NP-expressing vaccine might offer complete disease protection by generating intact but non-neutralizing antibodies. However, challenge experiments have demonstrated that GPC-expressing vaccination primarily induces a CD8 +T cell response and does not offer protection. Surprisingly, immunization of mice with vaccine candidates expressing both NP and GPC, even at very low doses, elicited the best control of viral multiplication and offered full protection from illness even after a high-dose challenge (36).

## 3 Current limitations

Understanding the pathophysiology of the virus and the development of a CCHFV vaccine has been severely limited by the absence of susceptible animal models for CCHFV infection that replicate the pathology of the virus typically encountered in humans. Previously, neonatal mice were the only vertebrates known to be susceptible to CCHFV in addition to humans (90). However, because these neonatal mice lack a mature immune response, there is no apparent progression of CCHFV symptoms as in humans (91). For this reason, the use of these animals as model organisms is limited. Animal model development for CCHFV has intensified since 2010 after it became clear that the suppression of type I interferon responses allowed CCHFV to cause lethal infection. The STAT1^−/−^mouse model utilized in the first of these experiments had a genetic deficiency in the STAT1 protein. Mice are susceptible to microbial infection but do not respond to IFN-γ or IFN-α. Although STAT1^−/−^mice are particularly susceptible to CCHFV infection, even a small dose can result in 100% mortality (92). IFNAR^−/−^ mice, homozygously negative for Type I interferon receptor I, are susceptible to CCHFV infection with Turkey04 and Ibar10200 strains, as opposed to wild-type mouse strains, and exhibit medical symptoms such as an acute viremia phase, high CCHFV RNA titers, and hepatomegaly, that are in line with the pathology observed in humans. Furthermore, infection of IFNAR^−/−^ mice with CCHFV also led to the development of coagulopathy and severe organ damage, similar to the observed effects in the STAT1^−/−^model. This accurately represents the hemorrhagic phase induced by CCHFV in humans (93, 94). A recent study by Hawman et al. (32), indicated that conducting vaccine development studies on mice with compromised immune systems may not accurately predict the vaccine’s effectiveness in humans. A DNA-based vaccine development study revealed that vaccine development studies in immunodeficient mice may not accurately predict the vaccine’s efficacy in humans (32). Moreover, a humanized mouse model was created by injecting CD34+ human stem cells into NSG-SGM3 mice (95) and when tested with CCHFV strains from Oman and Turkey, this humanized model exhibited distinct disease patterns. Fatal outcomes and neurological diseases were observed only with the Turkish strain. These mouse models hold significant potential as platforms for studying CCHFV pathogenesis and conducting preclinical analyses for vaccine development. The nonhuman primate (NHP) model, utilizing cynomolgus macaques, which closely resemble humans in terms of translational relevance, has also been developed (85). Initially, the model was characterized by up to 75% lethality; however, subsequent studies revealed that the severity of infection ranged from mild to moderate. Investigations using different CCHFV strains, such as Ibar-10200, Hoti, and Afg09-2990, demonstrated variable lethality in the macaque model (96, 97). This variability may be attributed to factors such as the outbred nature of macaques, variations in the virus strains used (e.g., passage number), and the age, origin, and low numbers of macaques used in the studies. Despite the observed variability in lethality, this animal model accurately represents a broad clinical spectrum for CCHFV similar to human infection. Therefore, until further research are completed or a more suitable alternative animal model is identified, cynomolgus macaques remain useful for the preclinical evaluation of anti-CCHFV therapeutics and vaccines. Animal model development efforts to study the pathogenesis of the CCHFV continue at great speed. However, due to the lack of a wild-type animal model that is both susceptible and immunocompetent for CCHFV and the fact that animal model development studies for CCHFV with transgenic methods have a relatively short history (approximately ten years). It is still necessary to further investigate the immune responses and mechanisms underlying these responses in these animal models. Vaccine development studies are expected to gain more momentum if a small rodent model is developed that closely mimics CCHFV symptoms in humans

The main structural components of the virus, the NP, and glycoproteins have been identified as potential antigenic targets for potential vaccines against CCHFV and have been used as vaccine antigens in almost all vaccine studies. Preclinical studies have shown protection in vaccines expressing CCHFV NP, GPC, or only glycoproteins Gn and Gc, suggesting a large number of protective epitopes within CCHFV. However, the identification of specific protective epitopes within these antigens is very challenging without immunoinformatic studies.

Glycoproteins Gn and Gc have commonly been regarded as the preferred antigens for the CCHFV vaccine since they are primarily localized on the surface of virus particles and are believed to play a role in eliciting neutralizing antibodies. There is, however, longstanding suspicion that M segment variability, particularly in the region encoding non-structural proteins, can affect cross-reactivity and ultimately negatively impact the ability to neutralize heterologous strains. By providing protection against genetically diverse strains of CCHFV, the vaccine could eliminate the further need for specific vaccines against evolving viruses. Because for the vaccine to provide complete protection, it is necessary to prevent the immune evasion of CCHFV, a virus with high genetic diversity. Although VSV-vectored and VLP-based CCHFV vaccines have achieved heterologous protection (22, 26), mice administered with a DNA-based vaccine expressing CCHFV GPC exhibited incomplete protection when challenged with a heterologous strain (30). However, in the preclinical evaluation of many vaccines, the use of only homologous strains in challenge studies is prevalent, and the extent of protection by these vaccines against other strains has not been fully elucidated.

The S segment of CCHFV encoding the NP is relatively more conserved between strains; therefore, CCHFV vaccines targeting NP have been shown to provide stronger protection, and it has been predicted that viral escape can be prevented by NP (25, 26, 32, 35, 36, 86). The mechanism linking vaccine-mediated protection against CCHFV to neutralizing antibody levels has yet been established. Notably, mRNA- and DNA-based vaccines have shown significant protection in mice or NHPs, failing to induce detectable levels of neutralizing antibodies following vaccination (25, 36, 85). In contrast, subunit- and VLP-based vaccines failed to protect mice exposed to a lethal dose challenge study, despite inducing high levels of neutralizing antibodies (23, 25). These data collectively suggest that, although humoral immunity is important, neutralizing antibodies are neither essential nor adequate for vaccine-mediated protection against CCHFV.

It should also be noted that the vaccine development platform used to construct a vaccine for CCHFV is essential. An MVA-vectored vaccine expressing the CCHFV NP failed to protect mice despite eliciting both humoral and cellular immunity (55), while an adenovirus-vectored vaccine also expressing the NP provided partial protection (3) and an mRNA vaccine expressing the NP provided 100% protection (35, 36).

Due to the perception that CCHFV is a virus that primarily affects the Middle East and particular countries in Asia and Africa, studies aimed at developing a CCHFV vaccine are more localized to the aforementioned regions rather than on a global scale, as was the case with SARS-CoV-2. While the identification of the virus dates back to the 1940s, CCHFV has been recognized as one of the infectious diseases most likely to cause major epidemic since 2015 (13). This contributes to the fact that the virus remains relatively new and unknown to the scientific community. Furthermore, most studies on CCHFV are carried out by research teams in institutes located in geographical areas where the virus is prevalent or within the framework of research projects such as Horizon of The European Union, where worldwide representation is limited. It is nonetheless necessary to establish, feed-forward, and sustain long-term, multi-national/multi-institute/multidisciplinary studies integrating immunoinformatic tools and *in vivo/in vitro* research to investigate both the virus’ pathophysiology and molecular structure, its immunological properties, etc., regardless of virus’ relatively limited geographical distribution. The conditions and factors outlined above contribute to the limitations of the developing vaccines for CCHFV and point to future directions for this field of research.

## 4 Current and future directions

Although many epidemics have been reported since the first appearance of the CCHFV, an effective vaccine against viral infection has not yet been established (2). Due to the fact that the genome of CCHFV contains mutations, mainly due to the error-prone polymerase segment, and the high rate of recombination in the RNA structure, the process of developing a vaccine to combat CCHFV can be quite challenging (6). In recent years, the design of multi-epitope-based vaccines against lethal viruses has gained considerable attention (98). Standard procedures used *in silico* vaccine design generally involve antigen selection, epitope prediction, vaccine re-engineering, and vaccine evaluation. However, antigenicity, allergenicity, toxicity, water solubility, hydrophobicity, and population coverage are considered crucial parameters during epitope predictions to improve the final designed structure. Suitable immunodominant epitopes and other structures are tandemly combined and separated by linker peptides for the final vaccine construct. Finally, the efficacy and stability of the vaccine are assessed through a range of bioinformatics approaches, including molecular docking, molecular dynamics, and *in silico* cloning (99). The first *in silico* vaccine design for CCHFV was published in 2019, and *in silico* vaccine design studies with potentially conserved multi-epitopes through an immunoinformatic approach have been conducted since then (100–104). However, *in vitro* or *in vivo* validation studies have not been performed for the designed multi-epitopes in a wet-lab environment. Evaluation of the efficacy and protection of vaccine candidates in a more realistic environment will significantly boost to develop a vaccine against CCHFV.

Current knowledge of the structure and function of CCHFV viral proteins continues to evolve in parallel with the development of new molecular biology tools and small animal models susceptible to CCHFV. Studies on host selection and disease mechanisms of CCHFV are also in progress. In particular, a more thorough understanding of the correlation between the protective efficacy of CCHFV vaccines and *in vitro* neutralization antibody levels will have the potentially lead to new vaccine design strategies that can induce more effective immune responses. All of these developments will require more interdisciplinary studies involving molecular biology, virology, veterinary health, bioinformatics, and protein engineering. The ability of glycoproteins to induce neutralizing antibodies and conserved regions of the NP to protect against heterologous strains brings the issue of the developing multiple epitope-based vaccines. On a parallel front, *in silico* molecular docking and immunoinformatic tools for epitope prediction of other structural proteins like RNA-dependent RNA polymerase encoded by the L segment may lead to new approaches for vaccine development.

Studies on the use of genetic adjuvants to enhance immune responses in CCHFV vaccine development have been limited to only a few recent studies (31, 33). With the resolution of the immune mechanisms of the virus, genetic adjuvants, which are promisingly used in vaccines developed against other viruses, may play a crucial role in increasing the protective efficacy of the CCHFV vaccine.

## Author contributions

Both BA and GBA contributed to the design and implementation of the review and the writing-editing of the manuscript. All authors contributed to the article and approved the submitted version.
